# Refining research outcomes: Lessons from the setup of an endpoint review committee and radiological review in tuberculosis observational diagnostic studies

**DOI:** 10.1371/journal.pgph.0006335

**Published:** 2026-04-30

**Authors:** Leyla Larsson, Claire J. Calderwood, Edson T. Marambire, Denise Banze, Alfred Mfinanga, Etienne Leroy-Terquem, Joseph Jacob, Daisuke Yamada, Francisco Trinchan Fernandez, Patrick Lungu, Anita Mesic, Ivan Noreña, Kathrin Held, Rishi K. Gupta, Lilian Tina Minja, Celso Khosa, Norbert Heinrich, Katharina Kranzer, On behalf of the ERASE-TB consortium

**Affiliations:** 1 Institute of Infectious Diseases and Tropical Medicine, LMU University Hospital, LMU Munich, Munich, Germany; 2 The Health Research Unit Zimbabwe, Biomedical Research and Training Institute, Harare, Zimbabwe; 3 Department of Clinical Research, Faculty of Infectious and Tropical Diseases, London School of Hygiene & Tropical Medicine, London, United Kingdom; 4 Instituto Nacional de Saúde (INS), Maputo, Mozambique; 5 National Institute for Medical Research- Mbeya Medical Research Centre, Mbeya, Tanzania; 6 Soutien Pneumologique International, Paris, France; 7 UCL Respiratory, University College London, London, United Kingdom; 8 Satsuma Lab, Hawkes Institute, University College London, London, United Kingdom; 9 Bulawayo City Health, Bulawayo, Zimbabwe; 10 East, Central, and Southern Africa-Health Community, Arusha, Tanzania; 11 Médecins Sans Frontières, Amsterdam, The Netherlands; 12 Unit of HIV & TB, Clinical Science Department, Institute of Tropical Medicine Antwerp, Antwerp, Belgium; 13 German Center for Infection Research (DZIF), Partner site Munich, Munich, Germany; 14 Fraunhofer Institute for Translational Medicine and Pharmacology ITMP, Immunology, Infection and Pandemic Research, Munich, Germany; 15 Departments of International Public Health and Clinical Sciences, Liverpool School of Tropical Medicine (LSTM), Liverpool, United Kingdom; 16 Department of Physiological Sciences, Faculty of Medicine, Eduardo Mondlane University (UEM), Maputo, Mozambique; Federal University of Rio de Janeiro, BRAZIL

## Abstract

Most tuberculosis diagnostic validation studies use microbiological or combination reference standards to define tuberculosis. We convened an Endpoint Review Committee (ERC) to define outcomes in ERASE-TB, a longitudinal cohort study across three high tuberculosis-burden countries, aimed at validating novel diagnostics for early detection. Herein, we describe processes and outcomes of the ERC. ERASE-TB enrolled 2,109 household contacts of people with tuberculosis who were followed up 6-monthly up to 24 months with clinical, microbiological, and radiological assessments at each visit. Any participants with a chest X-ray suggestive of tuberculosis or a positive Xpert MTB/Rif Ultra were investigated and eligible for endpoint review. For these, the clinical presentation, radiological, and microbiological results were reviewed independently by two study clinicians; any discordant endpoint categorisations were assessed by the ERC (first individually; if discordant, during a consensus meeting). Tuberculosis outcomes relied on predefined definitions (confirmed, likely, possible, unlikely). The ERC comprised four members: a tuberculosis programme manager, clinicians, and a radiologist. Semi-structured interviews (n = 4) with ERC members explored experiences and challenges in tuberculosis classification. A total of 96 clinical summaries underwent review, 55 were agreed internally, with the majority being categorised as confirmed tuberculosis (34/55, 61.8%). Among the remaining 41, the ERC members agreed on only 14 (34.1%) classifications, with the majority being classified as confirmed tuberculosis (10/14, 71.4%). The discordant 27 were discussed at the consensus meeting; 9/27 were classified as likely tuberculosis (33.3%), and 7/27 as possible and unlikely (25.9%). Qualitative interviews highlighted the complexity of tuberculosis diagnosis, value of longitudinal measurements, and tensions between decision making for research as compared to clinical purposes. Standardized tuberculosis classification frameworks, longitudinal data, and real-time expert review can improve diagnostic accuracy and comparability across studies. Future research should integrate structured ERC processes prospectively to refine tuberculosis case definitions and ensure robust clinical and research outcomes.

## Introduction

Tuberculosis remains the leading cause of death from a single infectious agent, with an estimated 10.9 million cases and 1.3 million deaths in 2023 [1]. Diagnosis remains difficult due to the disease’s complex pathophysiology and the limitations of current diagnostic tools [2]. The roll-out of rapid nucleic acid amplification tests has improved detection, but these rely on detectable levels of *Mycobacterium tuberculosis (Mtb)* DNA, which may be absent or insufficient in extrapulmonary tuberculosis or earlier states of disease [3], limiting sensitivity [4,5]. Mycobacterial culture is slightly more sensitive and often considered the “gold” standard, yet it too often fails to detect paucibacillary or early disease. Globally, in 2023, only 62% of tuberculosis notifications were bacteriologically confirmed, partially due to limited performance but also due to limited access to required diagnostic infrastructure [1]. While some people with clinically diagnosed, but unconfirmed, tuberculosis will truly have tuberculosis disease and benefit from treatment, others are misdiagnosed. Greater use of more sensitive diagnostics and repeated sampling, rarely done systematically in programmatic settings, would confirm more cases. However, in the absence of other diagnostic tools such as computed tomography (CT) scans and invasive sampling, an unknown proportion of people with unconfirmed tuberculosis are wrongly started on tuberculosis treatment.

The complex pathophysiology of tuberculosis, together with variability in the host immune response, leads to variability in disease progression, making accurate diagnosis based on single test at one time point challenging [6,7]. Intermittent bacterial shedding further increases the risk of missed detection from a single sample [8]. A proposed solution is the longitudinal follow-up of well-characterised participants with in-depth clinical investigations until a final diagnosis is achieved [9]. Based on this paradigm, we designed and implemented the ERASE-TB study, a prospective longitudinal observational cohort aimed at validating novel tests for earlier tuberculosis diagnosis [10]. To reduce bias, increase fidelity, and clearly define our study outcomes, we established an endpoint review committee (ERC) and external radiological review. The aim of this study was to describe the operationalisation and outputs of an ERC for tuberculosis outcome adjudication within a multi-country cohort, and to examine the methodological considerations of this approach, drawing on qualitative feedback from external reviewers to inform best practices for future tuberculosis diagnostics research.

## Methods

### Ethics statement

ERASE-TB was approved by regulatory and ethical committees of the participating institutions and national ethical committees: the Medical Research Council in Zimbabwe (MRCZ/A/2618), the National Health Research Ethics Committee in Tanzania (NIMR/HQ.R.8a/Vol.IX/3608), the National Bioethics Committee for Health in Mozambique (541/CNBS/21), the ethical committees of London School of Hygiene & Tropical Medicine, United Kingdom (22 522–2) and the medical faculty of the Ludwig Maximilian University, Germany (20–0771). Written informed consent was obtained from all participating adult household contacts. For minors (<18 years), written informed consent was provided by the parent or guardian, and assent was sought from the minor, according to local guidelines.

### Study population and aims

**Fig 1:**
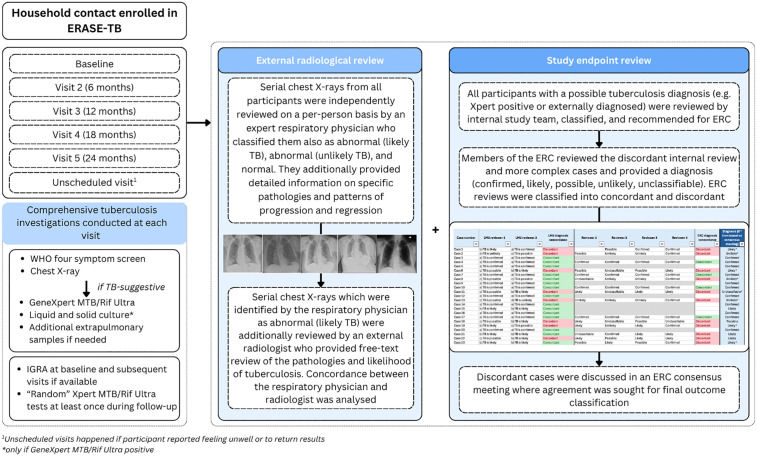
Processes undertaken in ERASE-TB to generate high fidelity research outputs. Abbreviations: ERC: endpoint review committee, WHO: World Health Organization, TB: tuberculosis, Xpert: Xpert MTB/Rif Ultra.

ERASE-TB was a longitudinal observational cohort study which recruited and followed-up 2,109 tuberculosis household contacts aged 10 years and above from 08/03/2021–06/12/2024 in Harare, Zimbabwe, from 12/08/2021–27/06/2025 in Maputo, Mozambique, and 02/09/2021–28/04/2025, in Mbeya, Tanzania, with the primary aim of validating novel diagnostics for early states of disease [10]. The household contacts were followed up on a 6-monthly basis for up to 24 months. At each visit, comprehensive tuberculosis screening was conducted which included symptom screening (modified WHO four symptom screen including cough of any duration; W4SS) and chest X-ray (CXR) which was read by a clinical officer who was part of the study team. If either was suggestive of tuberculosis, the household contact provided sputum for further investigation with Xpert MTB/Rif Ultra (Xpert MTB/Rif Ultra; Cepheid, USA). If the sputum sample tested positive for *Mtb* DNA, two additional sputum samples were taken and investigated with Xpert MTB/Rif Ultra and liquid (BACTEC Mycobacterial Growth Indicator Tube system, Becton Dickinson Microbiology Systems, USA), and solid culture (Löwenstein–Jensen) [10]. A participant was also able to i) conduct a study visit telephonically if in-person was not possible and ii) have an unscheduled visit in case of incident symptoms or concerns (Fig 1). Throughout the study, intermittent CXR and symptom agnostic Xpert MTB/Rif Ultra tests were performed, (i.e., non-systematic testing at all sites during follow-up), so that each participant had at least one microbiological test for tuberculosis during follow-up (between month 6 and 18; i.e., at Visit 2, 3, or 4). Once a participant reached eligibility for a CXR and symptom-agnostic test (i.e., a follow-up visit and screen negative), a sample was taken. Samples were not repeated at a later visit if a CXR and symptom-agnostic sample was already taken. Spontaneous sputum samples were stored at baseline and at the last visit for retrospective testing where needed. Stored baseline sputum samples were tested with Xpert MTB/Rif Ultra in all participants investigated for tuberculosis during follow-up.

All participants who had evidence of positive microbiology or CXR abnormalities were followed up in real-time with an in-depth tuberculosis investigation involving additional sample collection and additional CXR or CT scans where needed. In the case of external diagnoses, medical records were reviewed with participants’ permission and follow-up tests conducted where possible. A proforma containing a detailed clinical history, evolution of symptoms, and microbiological, radiological, and any other test results was completed for participants whose presentation triggered an in-depth real-time or retrospective investigation (supplementary materials). Any participant who had a proforma completed (through evidence of positive microbiology, CXR abnormalities, or external diagnoses) was first reviewed internally for endpoint classification.

### Endpoint definition and review

Two tuberculosis experts (NH, IN) at the Ludwig Maximillian University Hospital (LMU) served as internal reviewers (Fig 1). In parallel, an ERC was convened; including a senior physician with daily patient contact within the Zimbabwean national tuberculosis programme (FTF), an infectious disease physician with >15 years’ experience of treating people with tuberculosis across different high-tuberculosis burden countries (AM), a consultant radiologist specialised in respiratory disease with experience in the United Kingdom and several high-tuberculosis burden countries (JJ), and a public health specialist and previous Zambian national tuberculosis programme manager (PL). For each participant who required a review (internal and external), the reviewers received an anonymised package containing the proforma, serial CXR, and CT scans, if available. Reviewers also received an Excel spreadsheet with prespecified definitions of the disease classifications (Table 1) and were asked a series of questions related to type of tuberculosis (pulmonary/extrapulmonary), certainty of diagnosis (confirmed, likely, possible, unlikely, or unclassifiable), and date of diagnosis, including the earliest potential date tuberculosis could have been identified in retrospect (supplementary materials). The endpoint classifications were initially designed by the ERASE-TB study team and were reviewed and amended throughout the ERC review process (Table 1).

**Table 1:** Outcome definitions used by the endpoint review committee.

Classification	Definitions
Confirmed	Positive culture with speciation, Xpert MTB/Rif Ultra with highly infectious semiquantitative readout (i.e., high or medium); **EPTB with any microbiological evidence.**
Likely	Xpert MTB/Rif Ultra low/very low if symptoms also suggestive or CXR abnormal; **diagnosed externally with evidence of positive microbiology, EPTB presentation improving on treatment, EPTB presentation with imaging evidence, EPTB presentation followed by death likely due to TB.**
Possible	Single Xpert MTB/Rif Ultra trace triggered by symptoms, or diagnosed externally without available microbiological results.
Unlikely	CXR abnormal but not TB specific, Xpert MTB/Rif Ultra negative, or incidental Xpert MTB/Rif Ultra with single trace; **death attributable to TB reported by relative without supporting clinical/microbiological evidence, Xpert MTB/Rif Ultra trace or very low with no culture in the context of previous TB.**
Unclassifiable	Unable to classify into any of the above classifications due to insufficient data and information to meet the definitions.

Bold definitions are the clarifications which were added prior to the consensus meeting to support discussions and provide nuance to some of the complex cases. These additional definitions were reviewed and agreed by ERC members. All definition clarifications were made prior to the consensus meeting and before final endpoint adjudication. No changes were made after the consensus meeting or after outcome distributions were known to the investigators.

Abbreviations: EPTB: extrapulmonary tuberculosis, TB: tuberculosis, CXR: chest X-ray.

All cases were initially reviewed by the two internal reviewers. No further review was required if the internal reviewers agreed on each of disease classification, certainty, and date of diagnosis. In case of discordance, participant information was reviewed and classified by all ERC members.

The two-step review process was designed to balance feasibility with robust outcome classification. Initial assessment and classification were undertaken by senior study clinicians with extensive experience in TB clinical trials for cases with clear clinical and microbiological evidence. Cases with discordant findings or diagnostic uncertainty were subsequently referred to the multidisciplinary endpoint review committee (ERC). This approach ensured specialist adjudication was concentrated on diagnostically complex cases, where it provides the greatest added value.

Once feedback was collated from all ERC members a consensus meeting was organised to discuss discordant endpoints and reach a consensus outcome to be used for ERASE-TB research outputs. All members were invited, including the two internal reviewers, and the chair of the meeting (KK) presented each case and facilitated discussions. Majority voting was enough to establish consensus.

#### Semi-structured interviews.

Following the consensus meeting, semi-structured interviews were conducted with each of the members of the ERC with the aim of understanding the ERC process and refining this novel approach for use in future research. A topic guide was developed, exploring the ERC members’ experience conducting the review, inviting feedback on the format and content of the information provided and the review process itself, and inviting them to share learning experiences and suggestions for approaches to diagnostic classification of tuberculosis in future research. All interviews were conducted virtually over Zoom, audio-recorded, transcribed verbatim, and anonymised. Qualitative data were thematically analysed guided by a priori topics from the interview guide. Categories, sub-themes, and themes were then derived from the data, named, and iteratively refined.

### External radiological review

At the end of the study, and separately to the ERC, all CXR were reviewed by a consultant respiratory physician (ELT) with more than three decades of clinical experience in low- and high-tuberculosis burden settings. Serial CXR were provided, facilitating identification of progression and regression. The expert CXR review findings were documented in a standardised electronic case report form on OpenClinica software (Massachusetts, United States), capturing the presence of any abnormality, specific features (i.e., cavities, opacities, etc), and extrapulmonary involvement (supplementary materials). In addition, free-text summaries of findings were provided for participants who had any abnormality on any radiograph. The study team, including a clinical microbiologist (KK), three physicians with expertise in respiratory medicine, tuberculosis, and paediatrics (CJC, CK, DB), a nurse practitioner (ETM), and data analyst (LL), reviewed the free-text summaries and all available investigation results (clinical information, CXR, Xpert MTB/Rif Ultra and culture). Additional investigations (e.g., testing of stored sputum samples) were performed and participants were invited to attend the study clinic for clinical review, repeat CXR, and microbiological sputum investigations, where appropriate.

All CXR which were identified as abnormal, likely due to tuberculosis, were reviewed in a second stage by a consultant radiologist (DY), with special interest in respiratory disease, who provided a structured written report describing specific findings (Fig 1).

## Results

ERASE-TB recruited 2,109 household contacts (710 from Mozambique from 277 households, 699 from Tanzania from 277 households, and 700 from Zimbabwe from 268 households). The median age was 26.6 years (IQR: 16.7–41.8) and the majority were female (1,312/2,109, 62.2%). The median number of visits including unscheduled visits was 5 (IQR: 4–5), and 514 people were lost to follow-up or opted out during follow-up.

At any visit (total visits n = 8,395), 748 people (8.9%) reported tuberculosis-suggestive symptoms and 307 (3.7%) demonstrated tuberculosis-suggestive abnormalities on chest X-ray as per clinical officer review, resulting in 952 visits with a positive tuberculosis screen by 716 participants. Of these, 707/952 (74.3%) samples from 518 people (of 716 eligible) were investigated microbiologically, of which 89/707 (12.6%) had a positive Xpert MTB/Rif Ultra result (47/518 [9.1%] participants). The remainder did not have sputum samples collected or processed when the tuberculosis screen was positive. A total of 1,856 CXR and symptom-agnostic sputum samples (i.e., performed without the participant having symptoms suggestive of tuberculosis and without suggestive chest X-ray findings) from 1,498 participants were investigated with Xpert MTB/Rif Ultra. Of those, 74/1,856 (4.0%) were positive (38/1,498 participants).

**Fig 2:**
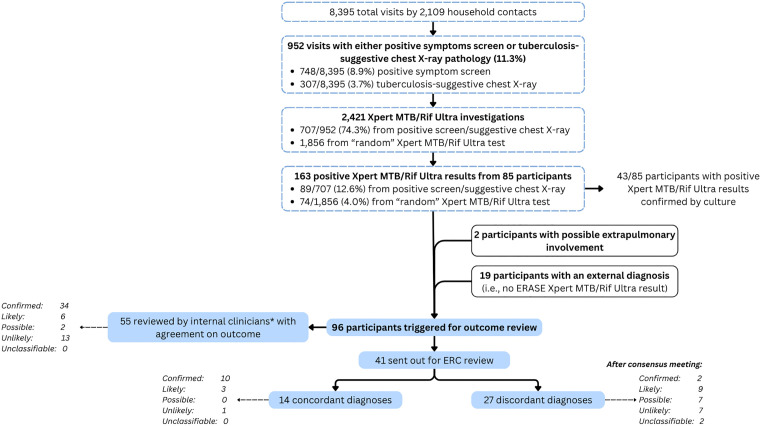
Flow chart of tuberculosis investigation and review process.

Of all positive Xpert MTB/Rif Ultra results (n = 163), 23 (14.1%) had a high semiquantitative readout, 15 (9.2%) medium, 43 (26.4%) low, 47 (28.8%) very low, and 35 (21.5%) trace. There were 145 cultures conducted from 77 participants; among these 43 people had positive cultures (76 positive culture samples) (Fig 2). A detailed breakdown of the participant microbiological investigations is found in S1 Fig.

### Endpoint review

The information of 96 participants underwent outcome review. Reasons for review were a positive Xpert MTB/Rif Ultra result (n = 73), clinical history or findings suggestive of extrapulmonary disease (n = 2), death (n = 2), or report of an external diagnosis (n = 19). Of participants undergoing review, 33 were from Mozambique, 28 from Tanzania, and 35 from Zimbabwe. Of these, 55/96 (57.3%) cases were reviewed and agreed upon by the two internal clinicians with the majority of those being categorised as confirmed tuberculosis (34/55, 61.8%) (Fig 2).

The information of the remaining 41 participants was sent for external ERC review. The independent review by ERC members resulted in 14 (34.1%) concordant classifications with the majority being classified as confirmed tuberculosis (10/14, 71.4%). 27 reviews were discordant, of which 18 were discussed during an online consensus meeting; for the remaining nine consensus was reached following repeat review using updated definitions agreed by the ERC members (Table 1). At the consensus meeting, most cases were classified as likely (9/27, 33.3%), followed by possible and unlikely tuberculosis (both 7/27, 25.9%) (Fig 2).

The entire review process ultimately resulted in 47 participants being classified as confirmed, 17 as likely, 9 as possible, and 21 as unlikely tuberculosis (2 were unclassifiable). Subsequent ERASE-TB analyses classified participants with either confirmed or likely tuberculosis as having tuberculosis (with sensitivity analyses using confirmed only as tuberculosis). 17/96 (17.7%) participants had positive Xpert Ultra results but were classified as possible or unlikely tuberculosis (of which 10 were trace-positive) (Table 2).

**Table 2:** GeneXpert MTB/Rif Ultra results by final ERC classification.

Classification	GeneXpert MTB/Rif Ultra results					Culture positivity
	Trace	Very Low	Low	Medium	High	
Confirmed	2	14	15	7	9	47
Likely	1	2	3	0	0	0
Possible	0	1	1	0	0	0
Unlikely	10	5	0	0	0	0
Unclassifiable	0	0	0	0	0	0

### Radiological review

Retrospective review of 5,719 CXR from 2,109 participants by an experienced consultant respiratory physician (ELT) identified abnormalities in 331 CXR from 275 participants, of which 242 (73.1%) CXR (190 participants) were suggestive of tuberculosis at some point during follow-up. 89/190 participants showed evidence of progressive pathology whilst 45/190 displayed undulating and/or regression of findings. CXRs of 34/190 participants were classified as being suggestive of TB sequelae. Classification into progression, regression/undulation, and sequelae was provided by ELT. These findings were reviewed by the study team (outlined above) who identified 60/134 participants (who showed signed of progression or regression/undulation) to be reviewed by a radiologist (DY). DY reviewed the CXR of the 60 participants blinded to the review conducted by ELT and provided their findings.

These radiological reports were concordant for 32/60 participants. In 24 participants, reports were discordant and reviewed by a third radiologist for adjudication. For 4 participants concordance could not be reached due to poor image quality.

#### Qualitative feedback.

All four ERC members participated in semi structured interviews conducted in English by LL (MSc, female) which lasted between 20–40 minutes. Thematic analysis identified three overarching themes: (i) difficulties in diagnosing tuberculosis, ii) characteristics of the members of the endpoint review committee, and iii) operationalisation of the review process (Table 2).

### Difficulties in diagnosing tuberculosis

All ERC members highlighted the challenges of accurately diagnosing tuberculosis given the limitations of current tools. They stressed that diagnosis requires synthesis of multiple sources of information (e.g., clinical, radiological, and microbiological) rather than relying on a single test result, underscoring the rationale for convening the ERC.

*“The only issue which limits the study, therefore, is what is obvious. The TB diagnostic tools are limited in diagnosing TB.”* (Member 3)

*“You wouldn’t have it another way, isn’t it, to assess that [diagnosing TB]? That is not how TB is done. TB is not black and white. You don’t make a diagnosis based on GeneXpert. You don’t make a diagnosis based on chest X-ray. You need to have a whole package of information.”* (Member 4)

Despite providing detailed clinical profiles, variability in tuberculosis progression complicated decision making. Some participants initially showed tuberculosis-suggestive features that later resolved, raising concerns about possible overtreatment if treatment decisions based on chest X-ray would be made at one single time point. In this context the longitudinal design of the ERASE-TB study was seen as a key strength.

*“And later, I was surprised that the patient after 2-3 years is not developing symptoms of TB and the patient is OK. So that has made me think as well; to think as well that maybe sometimes we are too aggressive starting TB treatment. It looks like there are a lot of questions and a lot of things we don’t know about TB.*” (Member 2)

*“The study brings out a very unique approach and starting to suggest that individuals who may have a presumptive TB call could be followed up longitudinally. Maybe by the same clinician or systematically using electronic medical record systems.”* (Member 3)

### Characteristics of endpoint review committee

None of the members had prior experience on an ERC and the novelty of the approach was a recurring theme, bringing specific challenges inherent to its discovery-based nature.

*“(…) it’s a really, really painful process, but I think until you do it, if what you’re doing is novel as opposed to just replicating what other people have done, it’s all discovery.”* (Member 1)

The primary aim of the review was to classify study participants using standardised definitions, as a definitive clinical case definition does not exist for tuberculosis classification.

*“Of course, you don’t want too many definitions out there because it’s just messy and confusing (…). The key things are that they have to be easy, they have to be quick, and they have to be reproducible. Your inter- and intra-observer variability have to be low.”* (Member 1)

Despite the study’s proposed definitions, considerable discordance remained among members classifying participants - largely due to differences in background and experiences. A key tension lay between the aims of research and clinical care; with ERC members noting that their prospective clinical judgement would have likely been different from this retrospective research-oriented analysis.

*“I think we could see the extreme variability for those challenging cases (…). One of them was trying to imagine exactly how this would work out in clinic, and not necessarily according to your variables, legend, and protocol. Whereas I was going purely, almost algorithmically on your variables and legend. So, we came to different conclusions.”* (Member 1)

*“I think it comes from where we come from, where did we work, what exactly we do with our job. I know that I am a person who would very easily give TB diagnosis and start TB treatment because I have seen so many missed cases ending up with more severe TB because we didn’t react in the beginning.”* (Member 4)

Despite their discordance members valued the diversity in experience and perspectives and praised the respectful communication within the group. At the same time, they acknowledged that aspects of “consensus-building” could feel somewhat artificial, highlighting the need for novel methods to achieve consensus.

*“That consensus process is always incredibly artificial because no one wants to be the one who is always saying they have the right opinion. (…) It’s not really a consensus of that individual case for the most likely diagnosis. I think there is a psychological overlay there. I’ve never found that consensus process realistic for consensing on an individual case. You end up just trying to be polite with the person next to you if you are a nice person.”* (Member 1)

### Operationalisation of the review process

**Fig 3:**
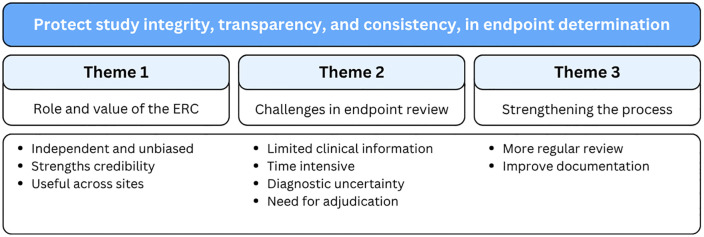
Themes and sub-themes identified through qualitative interviews.

Whilst members were generally satisfied with the ERC procedures, they identified areas for improvement, particularly i) clarity in instructions and information provided, ii) communication with members, and iii) the structuring and implementation of the review process. Suggested improvements included providing more comprehensive information in the proformas, conducting dry-run reviews, and involving members from the outset to build buy-in and foster a stronger sense of belonging to the study Fig 3.

*“Maybe what would be good for the future is that there is a meeting in person or online with the people who will review. It’s good to have these meetings and people can talk about “Ok, we will get these cases, it will be in the shape of an Excel, definitions will be here…” and I think that is a point of clarification before the process starts. It’s also good to do this in person rather than over email.”* (Member 4)

*“I think what would help is that, but it does require resources, is that the number of cases sent per review is smaller, and then you have a meeting afterwards where you discuss these discordances. And then people start to understand the context a bit better. They also hear the opinion of others. And it’s not that “oh I am going to be so influenced now, my replies are going to completely change”, but you also become a part of the study, not just like a totally passive observer somewhere thousands of kilometres away where you just reply to these cases.”* (Member 4)

## Discussion

Whilst tuberculosis diagnostics and therapeutics have progressed in recent years, meaningful progress in the early detection of tuberculosis especially paucibacillary, asymptomatic, and incipient tuberculosis remain limited by absence of robust reference standards particularly for disease states that may be intermittent and self-limiting [11]. In ERASE-TB, which aimed to evaluate novel diagnostics for earlier states of tuberculosis [10], we intended to address this challenge with longitudinal follow-up with serial CXRs and microbiological sampling, alongside the establishment an ERC to retrospectively classify participants according to predefined outcomes, thereby enhancing transparency and reproducibility. Operationalising this process highlighted the difficulty of establishing accurate tuberculosis diagnoses, even in well-resourced prospective research studies. Despite extensive clinical, microbiological, and radiological data collected across multiple time points classifying tuberculosis status remained challenging: among 96 participants reviewed, over 60% required adjudication beyond internal reviewers, and only one-third reached initial agreement by the ERC.

Crucially, ERC discordance was not random. Qualitative interviews showed that differences in members’ backgrounds, which included a public health officer, two tuberculosis clinicians, and one radiologist, shaped their interpretations. These differences reflected real-world tensions between clinical pragmatism (e.g., low thresholds for initiating treatment) and the strict adherence to predefined research criteria. Research settings allow for multiple samples and additional investigations with a substantially lower risk of losing patients to follow-up, enabling more certain diagnosis; an approach not feasible in resource-limited clinical practice. This tension was acknowledged by ERC members, with some noting that their clinical decisions would have differed from the retrospective academic classifications assigned in the study, highlighting the fact that this ERC process was specifically designed for research settings.

These reflections speak to a broader issue in research; the assumption that case definitions are well defined and easily applicable across settings. In reality, tuberculosis diagnosis requires synthesizing diverse streams of information including symptoms, imaging, microbiology, and particularly longitudinal changes [8,12–14]. Clinical features are further shaped by contextual factors, such as local disease burden, health system capacity, and socio-demographic characteristics (e.g., food insecurity, previous history of tuberculosis) [15,16]. Thus, a reliable diagnosis depends on the full clinical picture rather than a single test, raising the question of what should constitute a “minimal variable list” for establishing diagnosis of tuberculosis. This is critical not only for diagnostic validation studies, but also for vaccine trials. Paediatric tuberculosis research provides a useful precedent. Faced with diagnostic challenges due to the paucibacillary nature of disease and nonspecific symptomatology in children, an expert panel developed standardised clinical case definitions for use in research, encompassing socio-demographic and clinical characteristics [17–20]. This has enabled comparability across studies and pooled analyses. Whilst we aimed for similar consensus classifications in this study, heterogeneity of participant trajectories made this a complex, iterative process.

Lack of resources remains a prevailing theme across tuberculosis research. Longitudinal studies such as ERASE-TB are expensive yet yield relatively few diagnoses: of 2,109 participants enrolled, only 4% underwent full ERC review (4%) and just 3% were classified as confirmed or likely tuberculosis. This raises difficult questions about balancing comprehensiveness and feasibility. For example, systematic storage of induced sputum samples at every visit could have enabled retrospective diagnosis but was financially prohibitive. Still, the value of longitudinal investigation was clear from the radiological reviews: in several cases, serial CXR showed tuberculosis-suggestive pathology that later regressed, highlighting the heterogeneous natural history of tuberculosis [21,22]. Recent frameworks advocate for a spectrum-based model of tuberculosis, recognising early disease states between *Mtb* infection and microbiologically confirmed symptomatic tuberculosis disease (e.g., ICE-TB) [23,24]. Yet, practical case definitions and reference standards remain lacking - especially for ‘asymptomatic unconfirmed’ tuberculosis, where microbiology is negative. Rigorous expert review of longitudinal data, such as conducted in this study, can help provide empirical evidence to further refine such frameworks.

Strengths of this study include the wealth of prospectively collected clinical data and investigations which enabled in-depth retrospective assessment of most participants investigated for tuberculosis. Furthermore, the mixed methods approach provided detailed insights into the process of reaching consensus on outcome classifications. Limitations include the small number of participants with confirmed tuberculosis and that some participants were diagnosed outside of the study, resulting in limited investigations and samples in these cases.

Overall, these findings underscore the challenge of tuberculosis diagnosis even in adults, while highlighting the value of both longitudinal data and structured endpoint reviews in improving outcome standardisation for diagnostic and preventive studies. At the same time, the findings reveal the tensions between clinical and research priorities. Optimising tuberculosis diagnostic classification will require developing standardised definitions.

## Supporting information

S1 Fig Proforma filled in for in-depth investigation. (PNG)

S1 Text Questions asked to the ERC. **Table A:** Questions asked to the ERC. **Fig A:** Examples of investigatory trajectories of ERASE-TB participants highlighting the heterogeneity in TB progression.
(DOCX)

S1 Checklist Inclusivity in global researchresearch. (DOCX)
