# Supplementary figures and images for: Refining research outcomes: Lessons from the setup of an endpoint review committee and radiological review in tuberculosis observational diagnostic studies
Source: PLOS Glob Public Health. 2026 Apr 30;6(4):e0006335. doi: 10.1371/journal.pgph.0006335 (PMC13132429; doi:10.1371/journal.pgph.0006335)

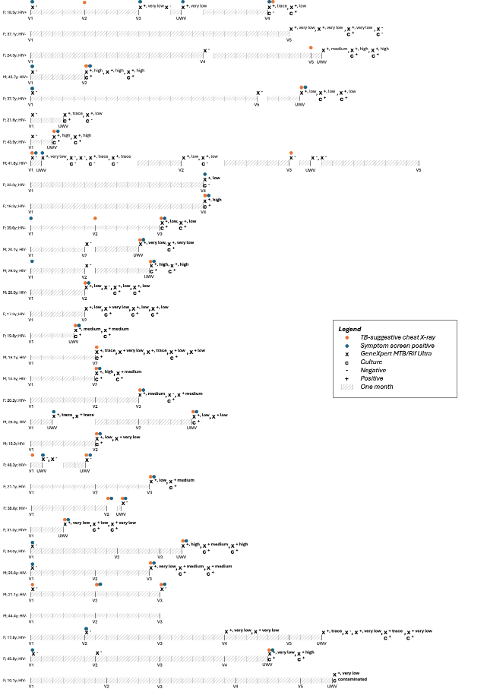

Supplement: S1 Fig — (PNG) [file pgph.0006335.s001.png]
