# Supplementary material for: Refining research outcomes: Lessons from the setup of an endpoint review committee and radiological review in tuberculosis observational diagnostic studies
Source: PLOS Glob Public Health. 2026 Apr 30;6(4):e0006335. doi: 10.1371/journal.pgph.0006335 (PMC13132429; doi:10.1371/journal.pgph.0006335)
Supplement: S1 Text — Table A: Questions asked to the ERC. Fig A: Examples of investigatory trajectories of ERASE-TB participants highlighting the heterogeneity in TB progression. (DOCX) [file pgph.0006335.s002.docx]

**Refining research outcomes: lessons from the setup of an endpoint review committee and radiological review in tuberculosis observational studies**

Table of Contents

[Supplementary materials 2](#_Toc186717307)

[TB-case proforma 2](#_Toc186717308)

[Questions asked to the ERC 4](#_Toc186717309)

## Supplementary materials

### Table A: TB-case proforma

| **PID number** |  | | **Sex** |  | | | | | | | | |
| --- | --- | --- | --- | --- | --- | --- | --- | --- | --- | --- | --- | --- |
| **Date of birth** |  | | | | | | | | | | | |
| **Study outcome** | **Completed study** | **TB diagnosed** | | | **Died** | | | | | | **LTFU** | |
| **VISITS** | | | | | | | | | | | | |
| **V1 date (DD/MM/YY)** |  | | **EOS date (DD/MM/YY)** | | | | | |  | | | |
| **V2 date (DD/MM/YY)** |  | | **In-person /telephonic** | | **Missed?** | |  | | | | |  |
| **V3 date (DD/MM/YY)** |  | | **In-person/ telephonic** | | **Missed?** | |  | | | | |  |
| **V4 date (DD/MM/YY)** |  | | **In-person /telephonic** | | **Missed?** | |  | | | | |  |
| **V5 date (DD/MM/YY)** |  | | **In-person /telephonic** | | **Missed?** | |  | | | | |  |
| **Total number of unwell visits** |  | | | | | | | | | | | |
| **PAST MEDICAL HISTORY** | | | | | | | | | | | | |
| **Date of HIV test at ERASE-TB** |  | | **Not applicable (Known HIV)** | | | | |  | | | | |
| **HIV history** (e.g. date of diagnosis, treatment regimens, CD4 counts) |  | | | | | | | | | | | |
| **TB history** (e.g. dates of previous TB episodes, any resistance, treatment completed or not) |  | | | | | | | | | | | |
| **Other past medical history** |  | | | | | | | | | | | |
| **Social and family history** |  | | | | | | | | | | | |
| **SYMPTOMS REPORTED DURING THE STUDY** | | | | | | | | | | | | |
| **Symptoms** | **Date first reported** | | **Any other dates reported / Notes** | | | | | | | | | |
| **Cough** |  | |  | | | | | | | | | |
| **Fever** |  | |  | | | | | | | | | |
| **Sweats** |  | |  | | | | | | | | | |
| **Weight loss** |  | |  | | | | | | | | | |
| **Other** |  | |  | | | | | | | | | |
| **TIMELINE TO TB DIAGNOSIS / DEATH** | | | | | | | | | | | | |
| At which visit was the first “suspicion” of TB raised -> e.g. symptom screen, abnormal CXR or positive Xpert? *If none, enter NA.* | | |  | | | | | | | | | |
| **Give a timeline of the events that led to the diagnosis of TB or death**  *Please use additional sheets as needed. If uncertain, enter approximate dates (e.g. month/year)* | | | | | | | | | | | | |
| **Date** | **Events** (e.g. Symptoms reported, CXR performed, Sample taken for Xpert, Sample taken for culture, Result of Xpert or culture received, Treatment given, TB diagnosed, TB treatment started) | | | | | | | | | | | |
|  |  | | | | | | | | | | | |
|  |  | | | | | | | | | | | |
| **Further details of clinical history (presentation, examination findings, treatment, investigations, including dates).** *Please use additional sheets as needed.* | | | | | | | | | | | | |
|  | | | | | | | | | | | | |
| **What was the cause of death (if known)?** |  | | | | | | | | | | | |
| **DIAGNOSTIC TEST RESULTS** | | | | | | | | | | | | |
| **Total sputum samples investigated** |  | | **Number of CXR performed** | | | | | | |  | | |
| **Number of samples tested with Xpert** |  | | **Number of samples tested with culture** | | | | | | |  | | |
| **Sample ID** | **Date (DD/MM/YY)** | | **Xpert result** | | | **Culture result** | | | | | | |
|  |  | |  | | |  | | | | | | |
|  |  | |  | | |  | | | | | | |
| **CXR - ID** | **Date (DD/MM/YY)** | | **Finding (clinical officer)** | | | | | | | | | |
|  |  | |  | | | | | | | | | |
|  |  | |  | | | | | | | | | |
|  |  | |  | | | | | | | | | |
|  |  | |  | | | | | | | | | |
|  |  | |  | | | | | | | | | |
| **Results of other diagnostic tests / tests performed outside ERASE-TB (e.g. CT scan, pleural tap, ultrasound, etc)** | | | | | | | | | | | | |
|  | | | | | | | | | | | | |

### Questions asked to the ERC

1. What is the certainty of TB diagnosis in this patient according to your view?
2. Is the TB pulmonary OR extrapulmonary?
3. If your answer to Q1 was that TB was likely or possible, what is the leading indicator here?
4. Would you classify this case of TB as “co-prevalent” or “incident”?
5. What is the first timepoint of possible detection of TB for this case
6. What is the timepoint that TB was present already, in retrospect?
7. if microbiology positive, was diagnosis by "for cause" or "incidental" testing?


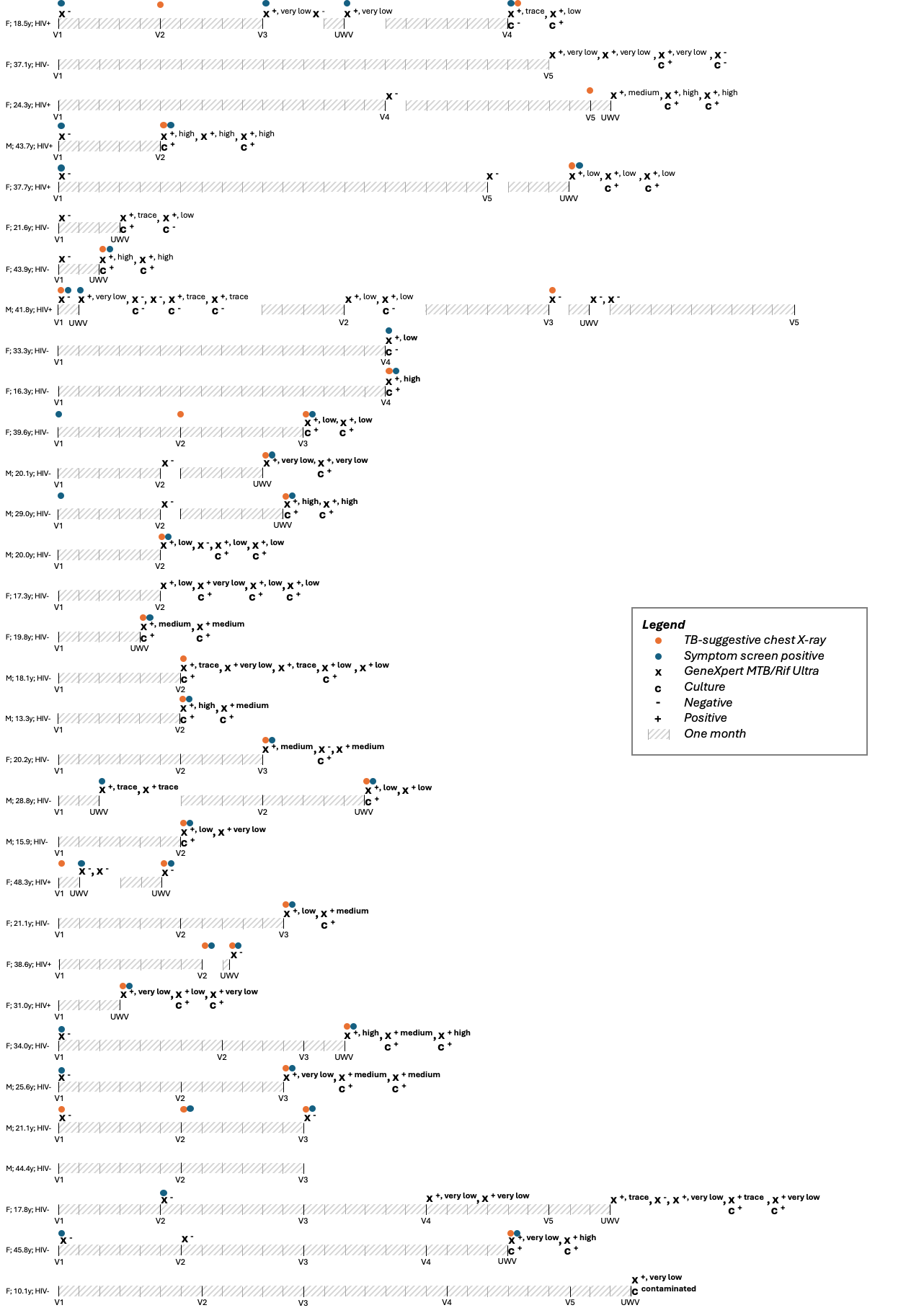


Fig A: Examples of investigatory trajectories of ERASE-TB participants highlighting the heterogeneity in TB progression
